# Directly observed social contact patterns among school children in rural Gambia

**DOI:** 10.1016/j.epidem.2024.100790

**Published:** 2024-12

**Authors:** Isaac Osei, Emmanuel Mendy, Kevin van Zandvoort, Olimatou Jobe, Golam Sarwar, Baleng Mahama Wutor, Stefan Flasche, Nuredin I. Mohammed, Jane Bruce, Brian Greenwood, Grant A. Mackenzie

**Affiliations:** a Medical Research Council Unit The Gambia at London School of Hygiene & Tropical Medicine, the Gambia; b Department of Disease Control, Faculty of Infectious and Tropical Diseases, London School of Hygiene & Tropical Medicine, London, UK; c Department of Infectious Disease Epidemiology, London School of Hygiene & Tropical Medicine, London, UK; d Centre for Mathematical Modelling of Infectious Diseases, London School of Hygiene & Tropical Medicine, London, UK; e Centre of Global Health, Charite – Universitätsmedizin, Berlin, Germany; f Murdoch Children’s Research Institute, Melbourne, Australia; g Department of Paediatrics, University of Melbourne, Australia

**Keywords:** School, Social contacts, Proxy-reported data, School-aged children, *Streptococcus pneumonia*

## Abstract

**Introduction:**

School-aged children play a major role in the transmission of many respiratory pathogens due to high rate of close contacts in schools. The validity and accuracy of proxy-reported contact data may be limited, particularly for children when attending school. We observed social contacts within schools and assessed the accuracy of proxy-reported versus observed physical contact data among students in rural Gambia.

**Methods:**

We enrolled school children who had also been recruited to a survey of *Streptococcus pneumoniae* carriage and social contacts. We visited participants at school and observed their contact patterns within and outside the classroom for two hours. We recorded the contact type, gender and approximate age of the contactee, and class size. We calculated age-stratified contact matrices to determine in-school contact patterns. We compared proxy-reported estimated physical contacts for the subset of participants (18 %) randomised to be observed on the same day for which the parent or caregiver reported the school contacts.

**Results:**

We recorded 3822 contacts for 219 participants from 114 schools. The median number of contacts was 15 (IQR: 11–20). Contact patterns were strongly age-assortative, and mainly involved physical touch (67.5 %). Those aged 5–9 years had the highest mean number of contacts [19.0 (95 %CI: 16.7–21.3)] while the ≥ 15-year age group had fewer contacts [12.8 (95 %CI: 10.9–14.7)]. Forty (18 %) participants had their school-observed contact data collected on the same day as their caregiver reported their estimated physical contacts at school; only 22.5 % had agreement within ±2 contacts between the observed and reported contacts. Fifty-eight percent of proxy-reported contacts were under-estimates.

**Conclusions:**

Social contact rates observed among pupils at schools in rural Gambia were high, strongly age-assortative, and physical. Reporting of school contacts by proxies may underestimate the effect of school-age children in modelling studies of transmission of infections. New approaches are needed to quantify contacts within schools.

## Highlights

• The validity and accuracy of proxy-reported contact data may be limited.
• Contact patterns were strongly age-assortative, and mainly involved physical touch.
• Overall, participants made an average of 17 contacts during the observation period.
• Those aged 5–9 years had the highest mean number of contacts.
• Proxy reporting was largely inaccurate compared to the observed school data.

## 1 Introduction

The extent of social contact between individuals plays an important role in the epidemiology and dynamics of several infectious diseases. During the COVID-19 pandemic, information on close contacts of cases played a significant role in understanding the effect of non-pharmaceutical interventions (Hellewell et al., 2020, Yuan and Blakemore, 2022, Verelst et al., 2021, Gimma et al., 2022). Most respiratory infections are directly transmitted from person to person through close contact, usually through respiratory droplets. Influenza virus, *Mycobacterium tuberculosis*, *Streptococcus pneumoniae,* and SARS-CoV-2 virus are examples of pathogens that are spread through close contact (Mossong et al., 2008, Edmunds et al., 2006, Feehan and Mahmud, 2021). Several studies have assessed the relationship between the epidemiology of respiratory infections and social contact patterns in different settings, particularly where high numbers of contact are made such as schools, places of worship, and work environments (McCaw et al., 2010, Beutels et al., 2006, Ibuka et al., 2016, Horby et al., 2011, Kiti et al., 2014, Melegaro et al., 2017).

During the COVID-19 pandemic, schools were designated as potential hubs for the spread of the infection. Consequently, many countries used school closures to limit the spread of infection, and schools remained closed for up to two years in some countries (Engzell et al., 2021). Several studies have identified school-aged children as the main driver of respiratory infections in various populations, (Mossong et al., 2008, Kiti et al., 2014, Qian et al., 2022) and social contacts between children and young adults are typically highly assortative compared to other age groups (Hoang et al., 2019, de Waroux et al., 2018).

A major challenge in estimating social contact patterns is the difficulty in precisely and accurately measuring patterns of proximal social interactions, especially in schools where a large amount of social mixing between individuals is anticipated (Brankston et al., 2007, Read et al., 2012, Grantz et al., 2021). In previous studies, parents and caregivers are used as proxies to estimate contacts of young children (typically those aged < 10 years) who are unable to complete questionnaires themselves (Hoang et al., 2019). The use of proxies to record contacts of young children, including school-going children, is a major limitation of several contact studies due to the potential inaccuracy of the reported data. Data verifying such proxy reports of school-based contacts are sparse (Toizumi et al., 2019). In a survey undertaken in Somaliland, parents or guardians who operated as proxies for their young children reported fewer contacts for their children at school, compared to contacts in households (van Zandvoort et al., 2022). A recent study by Hamilton et al. examined how potential errors in contact rates may bias model-based epidemic projections using an illustrative model of SARS-CoV-2 with two age groups (< 15 and ≥ 15 years). These researchers found that models with imbalanced contact rates underestimated the initial spread of SARS-CoV-2, had a later time to peak incidence, and a smaller peak incidence. They concluded that stratified transmission models that do not use accurate contact data may produce biased projections of epidemic trajectory and erroneous conclusions on the impact of public health interventions (Hamilton et al., 2024).

Various methods have been used to quantify social contacts including retrospective self-reports using interviews or surveys, and paper or digital diaries to report contacts prospectively (McCaw et al., 2010, Chen and You, 2015, Stein et al., 2015). The use of questionnaires and self-report methods may have several challenges, including the potential for recall bias, a low level of participation, and study fatigue, especially among highly active and mobile individuals such as school-age children (Mossong et al., 2008, Kiti et al., 2014, Smieszek et al., 2012). Recently, proximity-detecting wearable sensors (PDAs) have been used to collect data on social contact patterns, but these devices are unable to separate direct contacts from proximity or to capture certain contact characteristics such as physical touch, which is especially important in the transmission of certain organisms (Grantz et al., 2021, Fournet and Barrat, 2014).

Directly observing individuals and their interactions in time and space has been a useful method for studying human and primate social interactions (Kasper and Voelkl, 2009, Hamede et al., 2009). Direct observation allows the measurement of important contact details such as type of contact (physical or non-physical), duration, and frequency which could not be measured by other methods. Direct observation may be ideal for measuring contacts in small populations in closed settings, such as classrooms where all contacts can easily be observed (Read et al., 2012). Direct observation of an individual requires recording all contacts relevant to the transmission of infection. Direct observation offers a suitable method for capturing social contact information in young children who are unable to communicate clearly (Toizumi et al., 2019, Villaseñor-Sierra et al., 2007).

This study was part of a community-level social contacts survey to generate input data for mathematical modelling of interventions to influence pneumococcal transmission. In this paper, we describe school-based directly observed social contact and mixing patterns and assess the accuracy of proxy-reported compared to observed contacts in schools.

## 2 Methods

### 2.1 Study setting

**Fig. 1:**
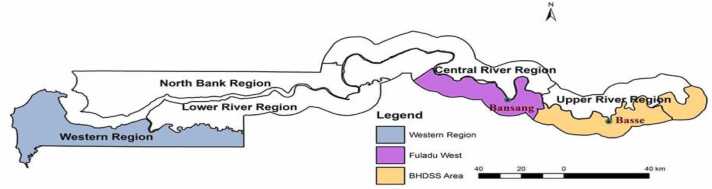
Map of the Gambia highlighting the areas where the Fuladu West and Basse Health and Demographic Surveillance Systems in Central and Upper River Regions operate.

The study was conducted in schools in the Central and Upper River Regions of The Gambia. The Gambia is a small country in West Africa with a population of 2.4 million. The Central River Region (CRR) and Upper River Region (URR) are in the east of The Gambia (Fig. 1). The Medical Research Council Unit The Gambia at the London School of Hygiene & Tropical Medicine (MRC Unit The Gambia at LSHTM) operates the Basse and Fuladu West Health and Demographic Surveillance Systems (BHDSS and FWHDSS) in URR and CRR respectively. In 2022, the BHDSS population was 206,429 (224 villages) and the FWHDSS population was 116,299 (217 villages); 15 % of the population was aged < 5 years. The annual birth cohort in the study area was approximately 8000.

#### 2.1.1 School system in The Gambia

The educational system of The Gambia comprises three years of early childhood and development, commonly referred to as nursery/kindergarten (ages 3–6 years); six years of lower basic education (ages 7–12 years); three years of upper basic education (ages 13–15 years); and three years of upper secondary education (ages 16–19). Most schools, especially those in rural settings, run a shift system in which some students attend classes in the morning and others in the afternoons. About 50 % of schools at primary and secondary level are government-supported. Non-government managed schools are categorized into Madrassas (Islamic or Qur'anic Memorisation schools), private schools and grant-aided schools (Barrow, 2022). As related to most schools in The Gambia, the majority of schools in the study area are mixed-gender, sharing the same classroom. There was only one single-gender school, which was all females.

#### 2.1.2 Approval for the study

Letters of approval from the Regional Education directorates were obtained and a copy of the approval letter and parental/participant written consent form was provided to the principals and class teachers of the schools of the selected students.

#### 2.1.3 Selection and enrolment of participants

The study aimed to recruit all school-going children aged 3–19 years, who had been enrolled in a household survey of nasopharyngeal carriage of *S. pneumoniae* and social contacts into the school-based contact survey. These participants were selected from 68 geographical clusters sampled from 441 villages using probability proportional to size. Field workers described the nature of the study to potential parents at the time of consent for the carriage survey. Efforts were made to ensure that the parent or caregiver understood the definition of contact by asking questions to confirm their understanding of the difference between physical and non-physical contact. During periods when a school was in session, efforts were made to conduct the school observations within a month from the day of the carriage survey. During school holidays, the observations were postponed until the school re-opened. A subset of the participants (18 %) were randomly selected to have their school observations performed on the same day that their parents/caregivers reported their contacts at school. The parents/caregivers were primed on the morning of the school observation and made aware that they would be contacted to report estimates of physical contacts at school. We visited 68 % of parents/caregivers at home or called 32 % by phone for their estimates of physical contacts for a full school day (Additional file 1).

#### 2.1.4 Data collection

Data collection was undertaken between 16 March and 14 December 2022. To ensure participants were blinded to the data collection process, trained field workers, different from those who obtained the consent, undertook the data collection. The field workers observed the participants in their classrooms for at least one hour and an additional one hour during the break period. Information on participants’ school grade, type of contact, gender and approximate age of contactees, and class size was recorded by the observer. Information on the least, average, and maximum age of students in the classroom was obtained from the class teacher to improve the estimation of the age of the contactee. The observers had no physical or verbal interaction with the selected participants during the observation period. The ratio of an observer to observee was 1:1.

Consistent with the definition used in the wider contact survey, we defined a contact as a two-way conversation between two people, occurring in a situation where pneumococcal transmission might be possible. A direct contact (contactee) was defined as an individual whom the participant met in person and with whom the participant had at least a short conversation and with whom they had either (i) “physical contact” (any sort of skin-to-skin contact e.g. a handshake, embracing, sharing a meal out of the same bowl, playing football or other contact sports, etc.”, or (ii) “non-physical contact” (did not touch the person but exchanged at least a few words, face-to-face within a two-metre distance). Multiple contacts with the same individual during the observation period were recorded as a single contact. If a contactor made both physical and non-physical contact with the contactee, physical contact was recorded.

Data were collected electronically using a custom-built electronic REDCap (Harris et al., 2009) application hosted at MRC Unit The Gambia at LSHTM.

### 2.2 Statistical analysis

We performed descriptive analyses of socio-demographic factors including school grade, class size, and contact information using simple frequencies and proportions for categorical variables and means, medians, and standard deviations for continuous variables.

Contact intensity was measured as physical or non-physical. We calculated age-stratified contact matrices to explore total (physical and non-physical) contact patterns and adjusted for reciprocity of contacts [i.e. the total number of contacts from age group *j* to age group *i* were equal to the total number of contacts from age group *i* to *j (m*_*ij*_*w*_*j*_ *= m*_*ji*_*w*_*i*_)] (Wallinga et al., 2006). Each matrix element (m_ij_) represented the estimated mean number of contacts between contactors in age group *i* with contactees in age group *j* and *w*_*i*_ indicates population size in age group *i*. Data on the number of contacts are presented using boxplots, histograms, and age-stratified contact matrices, created in R version 4.2.0. A mixed linear regression model including school as a random effect variable was constructed to explore the association between the mean number of observed contacts and selected socio-demographic factors. Age and sex were treated as a priori variables in the model. We measured the geometric mean contact, crude geometric mean contact ratios (the ratio of the mean number of contacts within each category of a variable relative to the reference category) and adjusted geometric mean contact ratios (adjusted for all socio-demographic variables in the model).

To assess the accuracy of parent/caregiver-reported estimated physical contacts at school compared to the observed data at schools, we estimated the mean ratio, degree of agreement, under-reporting or over-reporting. Data were analysed at the individual level, and we limited our comparative analysis to physical contacts to mainly focus on contacts relevant to the transmission of respiratory infections as shown in previous studies (Le Polain de Waroux et al., 2018, Neal et al., 2020). Differences in counts of physical contacts were generated by subtracting the parent/caregiver-reported estimated physical contact count from the school-observed physical contact count to indicate the level of agreement between the reported and observed counts. A negative count difference indicated overreporting by the parent/caregiver and a positive count difference indicated underreporting by the parent/caregiver. We measured the percent agreement within ± 1 count, ± 2 counts, ± 3 counts, ± 4 counts, and ± 5 counts with exact binomial 95 % confidence intervals (CI). Distributions of the reported and observed contact data were compared using a Kernel density plot. We performed a logistic regression analysis to examine the association between selected socio-demographic characteristics and agreement within ±2 counts. The outcome variable (agreement within ± 2 counts) in the regression analysis was treated as binary. A score of 1 was assigned to “agreement within ± 2 counts” and 0 to “disagreement”. We used Stata version 18.0 (StataCorp LLC, TX, USA) and R version 4.2.0 for all data management and analyses (Team, 2013).

### 2.3 Ethics and consent

The study was approved by the Gambia Government/MRC Joint Ethics Committee (ref: 28705) and by the LSHTM Ethics Committee (ref:28705). Written informed consent was obtained from parents or guardians of participants aged < 18 years and, in addition, assent was obtained from participants aged 12–17 years.

## 3 Results

### 3.1 Characteristics of the sample

We enrolled 219 participants from 114 schools. No families declined consent. The median age of the participants was 9 years (IQR: 7–13 years). There were slightly more female (53.4 %) than male (46.6 %) participants. About a fifth of the participants were in a nursery/kindergarten (20.5 %), 14 % were in secondary school, and 53.4 % were part of a class with more than 30 students. A total of 3822 contacts were registered. During the two hours of school observation, the median number of contacts was 15 (IQR: 11–20). The majority of the observed contacts occurred between those aged 5–14 years (74.4 % of all contacts). Most of the observed contacts were among lower basic school students (46.4 % of all contacts) and those with class sizes greater than 30 (64 % of all contacts; Table 1).

**Table 1:** Characteristics of participants and their observed contacts in school.

Characteristics	N (%)	Number of contacts N (%)	Median number of contacts (IQR)	Mean number of contacts (95 % CI)
Overall	219 (100)	3822 (100)	15 (11–20)	17.5 (16.2–18.7)
Age				
0–4 years	35 (16.0)	580 (15.2)	14 (9–21)	16.6 (13.4–19.7)
5–9 years	75 (34.2)	1425 (37.3)	16 (13–22)	19.0 (16.7–21. 3)
10–14 years	78 (35.6)	1421 (37.1)	16 (12–21)	18.2 (15.9– 20.5)
≥ 15 years	31 (14.2)	396 (10.4)	11 (9–17)	12.8 (10.9– 14.7)
Gender				
Male	102 (46.6)	1823 (47.7)	16 (12–21)	17.9 (15.2–18.9)
Female	117 (53.4)	1999 (52.3)	14 (10–20)	17.1 (16.2–19.6)
Grade				
Nursery/Kindergarten	45 (20.5)	801 (20.9)	15 (10–22)	17.8 (15.0–20.6)
Lower basic	94 (42.9)	1773 (46.4)	16 (13–21)	18.9 (16.7–21. 0)
Upper basic	49 (22.4)	730 (19.1)	14 (11–18)	14.9 (13.1–16.7)
Secondary	31 (14.2)	518 (13.6)	14 (9–20)	16.7 (12.6–20.8)
School Shift/Session				
Morning	147 (67.1)	2661 (69.6)	15 (11–22)	18.1 (16.4–19.8)
Afternoon	72 (32.9)	1161 (30.4)	15 (11–18)	16.1 (14.2–18.1)
Class size				
1–20	34 (15.5)	321 (8.4)	9 (7–11)	9.4 (8.3–10.5)
21–30	68 (31.1)	1053 (27.6)	13 (11–18.5)	15.5 (13.6– 17.4)
31–40	66 (30.1)	1316 (34.4)	17.5 (13–23)	19.9 (17.3–22.6)
> 40	51 (23.3)	1132 (29.6)	19 (16–30)	22.2 (19.7–24.8)
Contact Intensity				
Physical	NA	2579 (67.5)	10 (7–15)	11.8 (10.9–12.7)
Non-Physical	NA	1243 (32.5)	5 (4–8)	5.7 (5.1–6.2)

IQR – Inter quartile range.

95 % CI – 95 % Confidence Interval.

NA – Not Applicable.

#### 3.1.1 Mean number of contacts

During the two-hour observation period, the overall mean number of contacts was 17.5 (95 %CI: 16.2–18.7). Those aged 5–9 years had the highest mean number of contacts [19.0 (95 %CI: 16.7–21.3)] while the ≥ 15-year age group had the lowest mean number of contacts [12.8 (95 %CI: 10.9–14.7)]. The mean number of contacts increased with class size, from 9.4 (95 %CI: 8.3–10.5) in the smallest classes, to 22.2 (95 % CI: 19.7–24.8) in classes with > 40 children. There was no difference in the mean numbers of observed contacts by gender (Table 1).

#### 3.1.2 Contact Intensity

**Fig. 2:**
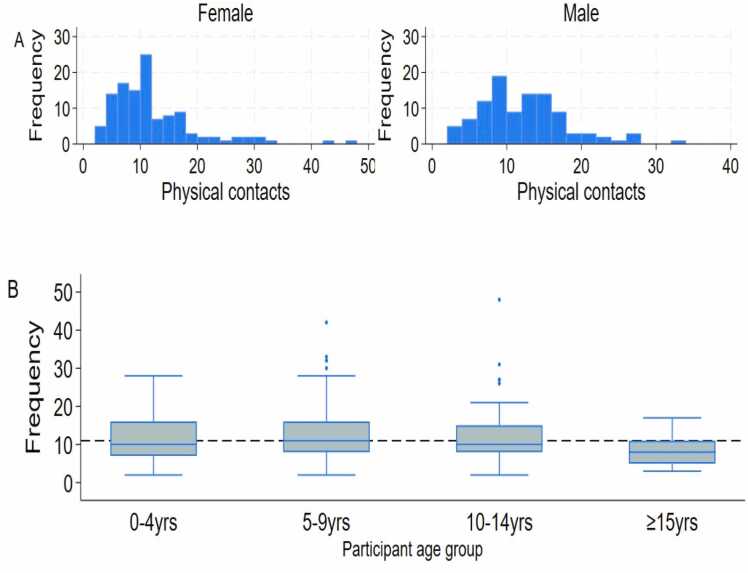
Distribution of physical contacts. Distribution of physical contact by participant gender (A), distribution of physical contact by participants age-group (B).

Contact intensity was measured as physical or non-physical. Two-thirds of contacts observed at schools were physical. Those aged 5–9 years had the highest mean number of physical contacts [13.1 95 %CI: 11.3–14.8)] while the ≥ 15 years age group had the lowest number of mean physical contacts [8.3 (95 %CI: 6.9–9.7)]. Both males and females had a similar mean number of physical contacts (Supplementary Table 1). Numbers of physical contacts by gender and age group are shown in Fig. 2.

#### 3.1.3 Gender-related contact pattern

**Fig. 3:**
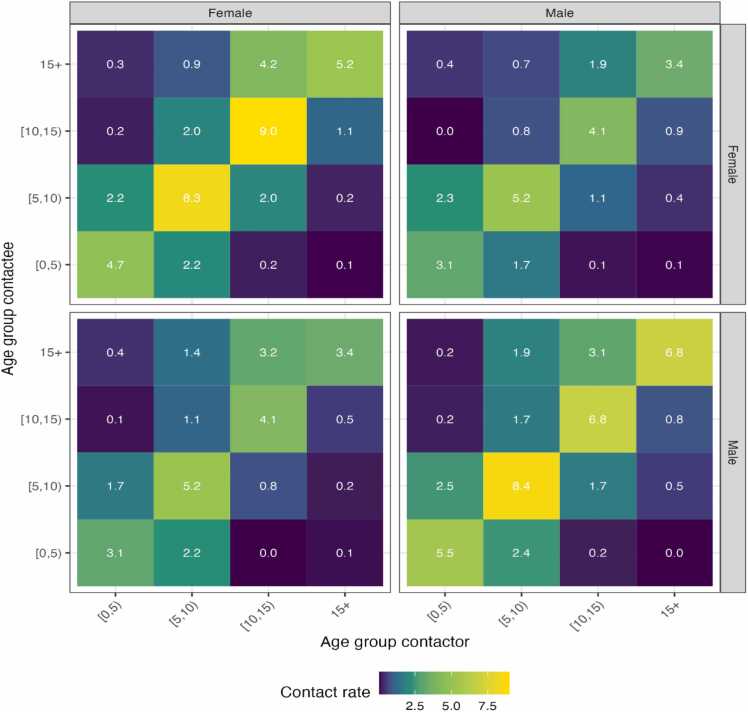
Contact matrices by gender mix. This shows the weighted mean number of daily contacts made by contactors of different gender and age groups with contactees of certain gender and age groups. Facet columns show the gender of the contactor, while facet rows show the gender of their contactees.

Similar age and same-gender interactions showed higher contact rates than different age and gender interactions, despite the lack of evidence of assortative contact by gender (Fig. 3).

#### 3.1.4 Age-specific contact matrices

**Fig. 4:**
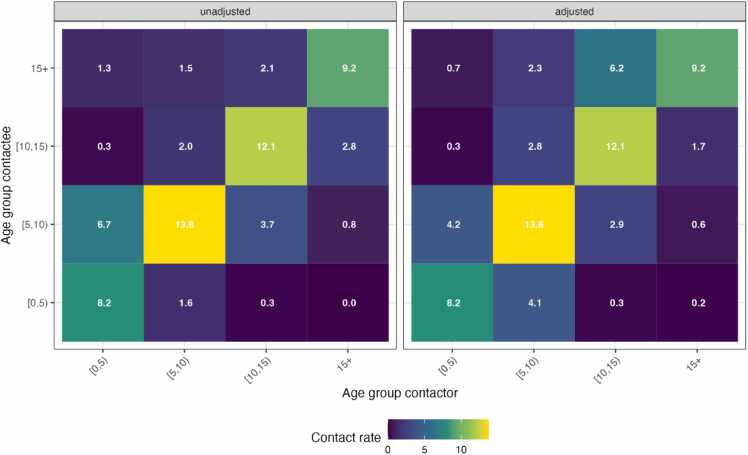
Contact pattern and matrices. Contact matrices show the mean number of observed contacts made by contactors with contactees of certain age groups (unadjusted), and age-specific estimates of mean contact rates adjusted for reciprocity of contacts.

The school contacts were highly age assortative as demonstrated by the high contact rates in the diagonal of the matrix. This indicates that participants were more likely to have contact with students in a similar age group. The highest mean contact rates were noted among the 5–9 and 10–14-year age groups. Both matrices show that mixing between age groups was low, except for 0–5 and 5–10 year-olds (Fig. 4).

#### 3.1.5 Factors associated with mean observed contacts

Class size was found to be significantly associated with the observed number of contacts. Those in classes of size > 40 students had more than double contacts [GMR 2.2 (95 % CI:1.8–2.7, p-value < 0.001)] contacts compared to those in classes with 1–20 students. There were no significant associations found between age group, gender, school grade, school shift, and the observed number of contacts (Table 2).

**Table 2:** Relative mean observed contacts at school, obtained from a mixed linear regression model.

Characteristics	N (%)	Geometric mean contact (95 %CI)	Crude GMR (95 % CI)	Adjusted GMR (95 % CI)	p-value (LRT)
Overall	219 (100)	15.40 (14.4–16.4)			
Age					
0–4 years	35 (16.0)	14.5 (12.2–17.2)	1 (ref)	1 (ref)	
5–9 years	75 (34.2)	16.9 (15.2–18.9)	1.2 (0.9–1.4)	1.1 (0.9–1.3)	0.42
10–14 years	78 (35.6)	16.0 (14.3–17.9)	1.1 (0.9–1.3)	1.1 (0.8–1.4)	
≥ 15 years	31 (14.2)	11.8 (10.2–13.6)	0.8 (0.6–1.0)	0.9 (0.7–1.3)	
Gender					
Male	102 (46.6)	16.0 (14.6–17.6)	1 (ref)	1 (ref)	
Female	117 (53.4)	14.9 (13.5–16.3)	0.9 (0.8–1.1)	0.9 (0.8–1.1)	0.41
Grade					
Nursery/Kindergarten	45 (20.5)	15.7 (13.5–18.2)	1 (ref)	1 (ref)	
Lower basic	94 (42.9)	16.6 (15.0–18.4)	1.1 (0.9–1.3)	0.9 (0.7–1.1)	0.24
Upper basic	49 (22.4)	13.8 (12.3–15.4)	0.8 (0.7–1.0)	0.80 (0.6–1.0)	
Secondary	31(14.2)	14.1 (11.5–17.3)	0.9 (0.7–1.1)	0.9 (0.7–1.2)	
School Shift/Session					
Morning	147 (67.1)	20.7 (20.3–21.1)	1 (ref)	1 (ref)	
Afternoon	72 (32.9)	17.9 (17.5–18.5)	0.9 (0.8–1.1)	1.0 (0.9–1.2)	0.62
Class size					
1–20	34 (15.5)	8.9 (7.9–10.0)	1 (ref)	1 (ref)	
21–30	68 (31.1)	13.9 (12.6–15.5)	1.6 (1.3–1.9)	1.5 (1.3–1.8)	< 0.001
31–40	66 (30.1)	17.9 (16.0–19.9)	1.9 (1.7–2.4)	1.9 (1.6–2.3)	
> 40	51 (23.3)	20.7 (18.7–22.9)	2.3 (1.9–2.8)	2.2 (1.8–2.7)	

LRT - Likelihood ratio test.

GMR - Geometric mean ratio.

95 %CI - 95 % confidence intervals.

#### 3.1.6 Accuracy of parent/caregiver reported contacts

Forty (18 %) of the 219 participants enrolled in the school-based contact study had their school-observed contact data collected on the same day that their parents/caregivers reported their school contacts. We limited our comparative analysis of these 40 participants to reported and observed physical contacts. Table 3 shows the mean ratio and percent agreement between parent/caregiver-reported and observed school physical contacts. The mean ratio between observed and parent/caregiver contacts was 1.1 (95 %CI: 0.8–1.3). More than half [57.5 % (95 %CI: 40.9–72.9 %)] of the parent/caregiver-reported contacts were under-reported while 40 % (95 %CI: 24.9–56.7 %) were over-reported. In a sensitivity analysis to assess the level of agreement within ± counts, the percent agreement within ± 1 count was only 12.5 %. However, this increased progressively to a percent agreement of 50.0 % at ± 5 counts. Thus, the difference in agreement between the parent/caregiver reported and observed contacts at school was wide even when the level of agreement was relaxed to a count of ± 5 (Table 3).

**Table 3:** Accuracy of parent/caregiver-estimated physical contacts at school compared to the observed data.

Indicator	Observed vs reported contacts percent (95 % CI)
Under-reporting	57.5 (40.9–72.9)
Over-reporting	40.0 (24.9–56.7)
Agreement within ± 1 contact	12.5 (4.2–26.8)
Agreement within ± 2 contacts	22.5 (10.8–38.4)
Agreement within ± 3 contacts	30.0 (16.6–46.5)
Agreement within ± 4 contacts	42.5 (27.0–59.1)
Agreement within ± 5 contacts	50.0 (33.8–66.2)
Mean ratio*	1.1 (0.8–1.3)

* Mean ratio of observed vs reported contact with 95 % CI.

**Fig. 5:**
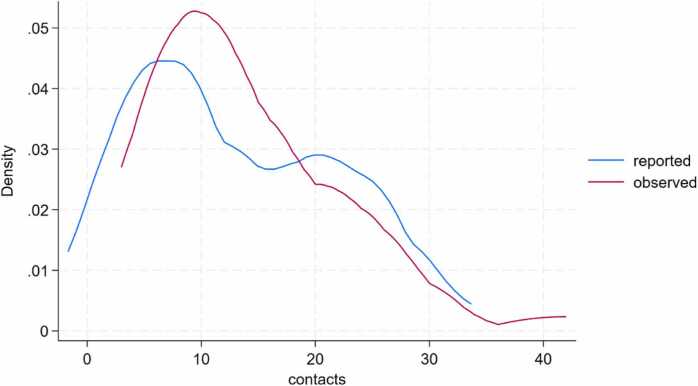
Kernel density plot showing observed versus proxy-reported contacts at school.

The Kernel density plot shows the density distribution in the observed data was relatively greater than that in the parent/caregiver reported data. This indicates underestimation in the parent/caregiver reported contact data compared to the observed data (Fig. 5).

There was no association between socio-demographic characteristics and agreement within ± 2 counts (Supplementary Table 2).

## 4 Discussion

Using the directly observed approach, we assessed the pattern of school-based social mixing contact among school children in rural Gambia. We also evaluated the validity of proxy-reported contact information by parents and caregivers versus school-based directly observed contact data. We found high observed mean contacts with strong assortativity by age. Proxy reporting was largely inaccurate compared to the observed school data. To our knowledge, this is the first directly observed school-based contact study that has assessed the accuracy of same-day proxy-reported contacts.

The mean number of 17.5 contacts observed in our study is comparable to that found in studies conducted among high school students in Taiwan (Chen and You, 2015, Luh et al., 2016), but lower than that noted in studies conducted among primary school pupils in Germany (32.7) (Mikolajczyk et al., 2008), secondary school students in the UK (70.3) (Jackson et al., 2011) and high school students in the US (29.0) (Leecaster et al., 2013). However, there are differences in the contact definitions and data collection methods between these studies and ours. While our study defined contact as a two-way conversation between two people occurring within two metres, these studies had a broader definition with no specified distance. Additionally, whereas our study used a directly observed approach, the studies mentioned above used various methods such as self-reporting, paper diary entries, and PDAs to quantify social contacts, emphasizing the significant role that contact definition, local setting and the type of data collection method plays in estimating contact patterns. Diary-based contact surveys have higher median daily contacts compared to other methods (Mousa et al., 2021). A recent systematic review of social contact surveys found that while a significant proportion of contacts in low- and middle-income countries occur at home, in contrast, the majority of contacts in high-income countries (HICs) occur at work or schools (Mousa et al., 2021). This might also explain the higher number of school contacts noted in HICs compared to the findings in our study. However, our observation period lasted only 2 hours as compared to some other studies in which students estimated their contacts over the full school day. We might have recorded more contacts in our study if we had made observations over a full school day.

The observed strong assortative age-mixing contacts in our study are similar to those reported in other studies (Kiti et al., 2014, Grantz et al., 2021, Fournet and Barrat, 2014). As expected, people of similar age groups tend to mix, especially in schools where classes are usually organized by age. Those aged 5–14 years had the highest contact rates among their age group. This was not surprising as children aged 5–14 years are highly active and more inclined to make physical contact with their friends. We observed a relatively high mixing between the 0–5 years and the 5–10-year age groups. This indicates the high potential for the spread of respiratory infection between these two younger age groups due to their high frequency of contact with each other. Similar to other studies, we observed that most contacts at school were physical (67.5 %) (Kiti et al., 2014, Fournet and Barrat, 2014, Thindwa et al., 2022, Potter et al., 2012, Mastrandrea et al., 2015). Although same-gender interactions showed higher contact rates than different-gender interactions, there was no evidence of assortative contact by gender. Class size was the only factor associated with higher contact rates with pupils in large-size classes being more likely to have higher contact rates than those in smaller-sized classes.

We evaluated the extent to which proxy (parent/caregiver) reported estimated physical contacts at school correspond to observed physical contacts collected for the same child on the same day. We found that proxy reporting of physical contacts often underreported compared to the observed data. Although various methods have been used to collect contact data to study infectious disease dynamics, few studies have examined the validity and accuracy of the data, especially data reported by proxies. Various studies have reported different outcomes when comparing different types of data collection tools. Few studies have sought to measure error and bias related to methods used to collect contact data. One such study, conducted by Smieszek et al. sought to evaluate how measurement error is related to diary methods. In their study, 50 participants from three research groups were asked to report contacts with other participants. The researchers found that for all matching pairs of contact reports, only 57.8 % of all contacts were recorded. They concluded that reporting error using the diary method was high and the probability of underreporting was higher for highly connected individuals compared to distant persons (Smieszek et al., 2012). A similar study by Smieszek et. al. directly compared the accuracy of reporting between an online-based survey and a sensor-based method among high school students in the United States. Students wore wireless sensor devices and also completed an online contact survey of their proximity conversational contacts for that day. The investigators collected the online-based and sensor-based data from the same setting and the same participant. They found 23.5 % concordance between the two methods and identified the online-based data as the primary source of underreporting (50 %). The researchers concluded that underreporting could be improved by excluding participants who reported few contact partners (Smieszek et al., 2014).

In our study, we found 57.5 % (95 % CI: 40.9–72.9 %) of parent/caregiver reports underestimated the number of contacts at school. The level of agreement increased modestly from 12.5 % to 22.5 %, and to 30.0 % when the definition of agreement was expanded within counts of − 1/+ 1, − 2/+ 2, and − 3/+ 3 respectively. Our results showed that the accuracy of proxy reporting was poor. Our finding of 57.5 % contact underestimation was higher than the 50.0 % reported by Smieszek et al. (2014). Besides the different settings and the methodological differences in data collection between the two studies, it is reasonable to assume that the true number of school-based contacts in our study would have been greater than that observed for the two hours had we recorded contacts for the six hours of the full school day.

Our study has several strengths. We recruited 219 participants from 114 different schools in the catchment area and our results can be generalised to the school population in rural Gambia. Additionally, we recruited participants from preschool (including nursery/kindergarten) and secondary schools and this allowed us to estimate contact rates within and between all ages of school-going children. The data collectors were unknown to the participants and had no physical nor verbal interaction with the selected participants during the observation period. This enabled observations to be made without a change in social behaviour by the participants. To our knowledge, this is the first directly observed contact study conducted within schools. This novel approach has provided insight into social mixing patterns in a setting that is difficult to assess. No COVID-19 control measures, such as wearing masks or social distancing, were observed during the survey period.

There are some important limitations to our study. First, the investigators spent only two hours at the school during data collection (one hour in the classroom and one hour during break time). Students usually spend about six hours at school and contacts may have been missed during the time of non-observation. Thus, the number of contacts that we observed will underestimate the true number of contacts during one full school day. Secondly, although our target sample size of school-going children was 272, we noticed that approximately 15 % of children of school-going age did not attend school. Additionally, some school-going children were absent from school during the survey day as they were sent to the farm during ploughing and harvesting seasons.

In rural settings, a high number of social contacts may occur while school children trek on their way to and from the school. Most rural settings have large neighbouring compounds and children usually engage in pre- and post-school activities where frequent social contact may occur. These out-of-school contacts could have an important influence on respiratory pathogen transmission. We were unable to observe such pre-school and post-school contacts. Our findings may not be generalised to urban settings in The Gambia where class sizes are generally smaller. The sample size of 40 to assess the validity of proxy-reported contacts versus directly observed data was limited. We assumed that a sample size of between 10 % and 20 % of the enrolled participants would be sufficient for the validation exercise. Due to our relatively small sample size, we were unable to assess whether the discrepancy between the observed and reported contact could have been affected by factors such as the student's age. Additionally, we did not assess proxy-reported contacts versus reported contacts from older children. Future studies could assess the influence of students' age on the accuracy of self-reported contacts to proxy-reported contacts.

## 5 Conclusion

Social contact at schools is very frequent, physical, and strongly age-assortative in rural Gambia. School-going children are potentially important drivers for the transmission of respiratory pathogens in their households and communities. Proxy contact reporting is largely inaccurate and generally underestimates the frequency of contacts. Though contact data at schools and in other closed spaces may be difficult to assess, especially in rural settings, and among young children, novel approaches should be employed to better quantify these contacts rather than relying on proxy reporting.

## CRediT authorship contribution statement

**Nuredin I Mohammed:** Writing – review & editing, Supervision, Formal analysis, Data curation. **Jane Bruce:** Writing – review & editing, Supervision, Investigation. **Brian Greenwood:** Writing – review & editing, Supervision, Methodology, Investigation, Conceptualization. **Grant A Mackenzie:** Writing – review & editing, Validation, Supervision, Resources, Investigation, Funding acquisition, Conceptualization. **Olimatou Jobe:** Writing – review & editing, Methodology, Data curation. **Golam Sarwar:** Writing – review & editing, Supervision, Methodology, Investigation, Data curation. **Baleng Mahama Wutor:** Writing – review & editing, Supervision, Methodology, Investigation. **Stefan Flasche:** Writing – review & editing, Investigation, Conceptualization. **Kevin van Zandvoort:** Writing – review & editing, Supervision, Investigation. **Isaac Osei:** Writing – review & editing, Writing – original draft, Investigation, Formal analysis, Data curation, Conceptualization. **Emmanuel Mendy:** Writing – review & editing, Methodology, Investigation, Data curation.

## Funding

The work was supported by the 10.13039/100000865Bill and Melinda Gates Foundation as part of the Pneumococcal Vaccine Schedule (PVS) trial. The funders had no role in study design, data collection and analysis, decision to publish, or preparation of the manuscript.

## Abbreviations

BHDSS: Basse Health and Demographic Surveillance System

FWHDSS: Fuladu West Health and Demographic Surveillance Systems

PVS: Pneumococcal Vaccine Schedules trial

MRCG: The Medical Research Council Unit The Gambia

## Declaration of Competing Interest

The authors declare that they have no known competing financial interests or personal relationships that could have appeared to influence the work reported in this paper.

## Data Availability

Anonymized data and questionnaire scripts are available on Zenodo via 〈https://doi.org/10.5281/zenodo.11428980〉.
