# Supplementary material for: Directly observed social contact patterns among school children in rural Gambia
Source: Epidemics. 2024 Dec;49:100790. doi: 10.1016/j.epidem.2024.100790 (PMC11649533; doi:10.1016/j.epidem.2024.100790)
Supplement: Supplementary file 1 — Supplementary material. [file mmc1.docx]

**Supplementary Table 1: Mean number of observed physical contacts at school.**

| **Contact measures** | **Total contacts**  **N (%)** | **Mean (Standard Deviation)** | **95% Confidence Interval** |
| --- | --- | --- | --- |
| Total contacts | 3822 | 17.5 (9.6) | 16.2 - 18.7 |
| Intensity |  |  |  |
| Physical | 2579 (67.5) | 11.77 (6.9) | 10.8 - 12.7 |
| Non-physical | 1243 (32.5) | 6.03 (3.9) | 5.5 - 6.6 |
| Physical Contacts | | | |
| Age |  |  |  |
| 0-4 years | 407 (15.8) | 11.6 (6.4) | 9.4 - 13.8 |
| 5-9 years | 980 (38.0) | 13.1 (7.6) | 11.3 - 14.8 |
| 10-14 years | 935 (36.2) | 12.0 (7.1) | 10.4 - 13.6 |
| ≥15 years | 257 (10.0) | 8.3 (3.8) | 6.9 - 9.7 |
| Gender |  |  |  |
| Male | 1192 (46.2) | 11.7 (5.9) | 10.5 - 12.8 |
| Female | 1387 (53.8) | 11.9 (7.8) | 10.4 - 13.3 |
| Grade |  |  |  |
| Kindergarten | 552 (21.4) | 12.3 (6.7) | 10.2 - 14.3 |
| Lower basic | 1216 (47.1) | 12.9 (7.9) | 11.3 - 14.6 |
| Upper basic | 484 (18.8) | 9.9 (4.8) | 8.5 - 11.3 |
| Secondary | 327 (12.7) | 10.5 (6.3) | 8.2 - 12.9 |
| School Shift/Session |  |  |  |
| Morning | 1816 (70.4) | 12.3 (7.4) | 11.1 - 13.6 |
| Afternoon | 763 (29.6) | 10.6 (5.8) | 9.2 - 11.9 |
| Number in class |  |  |  |
| 1-20 | 208 (8.1) | 6.1 (3.0) | 5.1 - 7.2 |
| 21-30 | 705 (27.3) | 10.4 (5.6) | 9.0 - 11.7 |
| 31-40 | 893 (34.6) | 13.5 (7.9) | 11.6 - 15.5 |
| >40 | 773 (30.0) | 15.2 (6.5) | 13.3 - 16.9 |
