# Supplementary material for: Directly observed social contact patterns among school children in rural Gambia
Source: Epidemics. 2024 Dec;49:100790. doi: 10.1016/j.epidem.2024.100790 (PMC11649533; doi:10.1016/j.epidem.2024.100790)
Supplement: Supplementary file 2 — Supplementary material. [file mmc2.docx]

**Supplementary Table 2**

**Factors associated with agreement within -2/+2 count, obtained from a multivariable logistic regression model.**

| **Characteristics** | **Crude Odds Ratio (95% CI)** | **P value** |
| --- | --- | --- |
| Age |  |  |
| 0-4 years | 1 (ref) |  |
| 5-9 years | 0.6 (0.1-4.5) | 0.60 |
| 10-14 years | 0.6 (0.1-5.2) | 0.61 |
| ≥15 years | 1.3 (0.12-13.24) | 0.08 |
| Gender |  |  |
| Male | 1 (ref) |  |
| Female | 1.4 (0.30-6.9) | 0.64 |
| Grade |  |  |
| Nursery/Kindergarten | 1 (ref) |  |
| Lower basic | 0.3 (0.0-1.8) | 0.17 |
| Upper basic | 0.3 (0.1-2.4) | 0.28 |
| Secondary | 0.4 (0.0-4.7) | 0.45 |
| School Shift/Session |  |  |
| Morning | 1(ref) |  |
| Afternoon | 2.3 (0.5-10.7) | 0.29 |
| Number in class |  |  |
| 1-20 | 1 (ref) |  |
| 21-30 | 0.2 (0.0-1.7) | 0.14 |
| 31-40 | 0.1 (0.0-1.5) | 0.09 |
| >40 | 0.1 (0.0-1.1) | 0.06 |
