# Supplementary material for: Directly observed social contact patterns among school children in rural Gambia
Source: Epidemics. 2024 Dec;49:100790. doi: 10.1016/j.epidem.2024.100790 (PMC11649533; doi:10.1016/j.epidem.2024.100790)
Supplement: Supplementary file 3 — Supplementary material. [file mmc3.pdf]

49.1.	Date of Screening	CSDATE	DD/MM/YYYY; autogenerate, editable	*SDATE_PCS*
49.2.	Participant First Name	CSPTFNM	Free text; *mandatory*	*PTFNM_PCS*
49.3.	Participant Last Name	CSPTLNM	Free text; *mandatory*	*PTLNM_PCS*
49.4.	DSS Resident	CSDSSRES	0=No, 1=Yes; *mandatory*	*DSSRES_PCS*
49.5.	DOB	CSDOB	DD/MM/YYYY; *mandatory, include unknown tick box generates 01/01/1900.*	*DOB_PCS*
49.6.	Age	CSAGE	01-99, *Autogenerated from DSS, OR activated for manual entry if =01/01/1900.*	*AGE_PCS*
49.7.	Age Strata	CSAGESTRATA	1=Months, 2=years, *Autogenerated from DSS OR activated for manual entry if =01/01/1900.*	*AGESTRATA_PCS*
49.8.	Age Category	CSAGECAT	1=0-11mo, 2=12-23mo, 3=24-59mo, 4=5-14yrs, 5=15-44yrs, 6=>=45yrs; *autogenerate, editable*	*AGECAT_PCS*
49.9.	Village Name	CSVILL	70001-79999 if 1.6=2, 80001- 89999 if 1.6=1; *mandatory Dropdown with Village names & numbers*	*VILL_PCS*
49.10.	Village Code	CSVCODE	*70001-79999 if 1.6=2, 80001- 89999 mandatory;* Dropdown with Village codes	*VCODE_PCS*
49.11.	Compound Number	CSCMPNUM	List, restricted, plus 'non-DSS'; from DSS; *mandatory*	*CMPNUM_PCS*
49.12.	Household Number	CSHHNUM	List, restricted, plus 'non-DSS'; from DSS; *mandatory*	*HHNUM_PCS*
49.13.	Participant DSSID	CSPTDSSID	5-14 digits, restrictions, may include suffix 'P'; from DSS; mandatory	*PTDSSID_PCS*
49.14.	Sex	CSSEX	Male=0, Female=1; mandatory; from DSS	*SEX_PCS*
49.15.	Ethnicity	CSETHCITY	0=Serahule 1=Fula 2=Mandinka 3=Wolof 4=Jola 5=Other; *mandatory*	*ETHCITY _PCS*
49.16.	If Eligible, verbal consent?	CSGOTOCONS	0=No, 1=Yes; -66=Skip	*GOTOCONS_PCS*
49.17.	Form End Date	CSEDATE	DD/MM/YYYY; autogenerate	*EDATE_PCS*
49.18.	Form End Time	CSETIME	HH:MM; autogenerate	*ETIME_PCS*
49.19.	Staff Code	CSPERSID	3 Digits, restriction PCS field staff	*PERSID_PCS*

	Activate if 50.5 =1		
55.1. Date of interview	CSCDATE	DD/MM/YYYY*; autogenerate, editable*	*_DATE_CSC*
55.2. PCS Study ID	CSID	PCS- [6 DIGITS] [D/E]; *Autofill from 50.9*	*ID_CSC*
55.3. Day of the week of the interview	CSCDYOFWK	1= Monday,2= Tuesday, ,3= Wednesday, 4= Thursday, 5= Friday, 6= Saturday, 7= Sunday, *mandatory*	*DYOFWK_CSC*
55.4. How many residents live in your household, including yourself? (Over the past two weeks)	CSCNHHRES	0-99, *mandatory*	*NHHRES_CSC*
55.5. Household member 1 Name	CSCHHMNM	*Auto filled from DSS Resident episodes table, Free text editable*	*HHMNM_CSC*
55.6. Household member 1 DSSID	CSCHHMDSSID	*Auto filled from DSS Resident episodes table, 5-14 Digit number, editable*	*HHMDSSID_CSC*
55.7. Household member PCS ID	CSCSTID	PCS]-[1/2][1/2/3][0-9999][D/E], (tick boxes, Not applicable=-88 for non-participants, Unknown=-99 for potential study participants without ID generated yet).; *mandatory*	*STID_CSC*
55.8. Household member 1 Sex	CSCHHMSEX	0=Male, 1=Female, *Auto filled from DSS Resident episodes table, mandatory editable*	*HHMSEX_CSC*
55.9. Household member 1 Age	CSCHHMAGE	0-99*; Auto filled from DSSID Individual mandatory editable*	*HHMAGE_CSC*
55.10. Household member 1 Age Strata	CSCHHMAGSTR	0=Months 1=Years; *Auto filled from DSSID Individuals, mandatory editable*	*HHMAGSTR_CSC*
55.11. Household member 1 Relationship	CSCHHMREL	1=Me, 2=Spouse, 3=Sibling, 4=Child, Parent, 6=Grandparent, Uncle/Aunt=7, 9=Other Family, 9=Unrelated, *mandatory.*	*HHMREL_CSC*
REPEAT, 55.8-55.14 based on auto filled form household members from 55.7, using the resident episode table. Include method to Add/remove members no longer there or not included from resident episode tables. 2.6 Should equal total number of			

household members			
REPEAT 55.17-55.20 dependent on how many members listed in 55.16.			
How often you/your child travel outside of your village or town and for how often?			
To a village/town <5km (<1-hour walk)			
55.12. How often do you/your child travel to those places outside of your village?	CSCFQNRTRV	1=Most days of the week, 2= At least once a week, 3= At least once a month, 4= less than once a month, 6=Never -99= Unknown, *mandatory*	*FQNRTRV_CSC*
55.13. How long do you/your child spend in that place when you/they go?	CSCNRTRTME	1=<1hr, 2=1-2hrs, 3= Half a day, 4= At least a whole day, *Mandatory but Not-activated if 55.12=6*	*NRTRTME_CSC*
To a village/town 5km (>1-hour walk)			
55.14. How often do you/your child travel to those places outside of your village?	CSCFQFARTRV	1=Most days of the week, 2= At least once a week, 3= At least once a month, 4= less than once a month, 6=Never -99= Unknown; *mandatory*	*FQFARTRV_CSC*
55.15. How long do you/your child spend in that place when you go?	CSCFARTRTME	1= At least a whole day, 2=Half a day, 3=1-2hrs, 4=<1hr; Mandatory *but Not-activated if 55.14=6*	*FQFARTRV_CSC*
55.16. Name of the furthest town you/your child visited last week?	CSCNTWNTRV	1=Didn't travel anywhere+ +Dropdown of DSS village list+ Outside CRR/URR+ Outside Gambia+ Other, *mandatory.*	*NTWNTRV_CSC*
55.17. If other, please specify.	CSCOTHTWNTRV	Free text, *mandatory if 55.16=Other*	*OTHTWNTRV_CSC*
Where did you/your child spend time yesterday?			
55.18. Type of place 1	CSCTYPOFPLC	1=Home, 2= Another House, 3=Work, 4=School, 5=Place of worship, 6=Public transport, 7=Market, 8= Field/Garden, 9=Other, *mandatory [allow multiple answers]*	*TYPOFPLC_CSC*
55.19. Type of place 1, if other please specify.	CSCOTHPLC	Free text, *activated & mandatory if 4.27=9*	*OTHPLC_CSC*
55.20. Time spent in place 1.	CSCTMSPNT	1=<15mins, 2=<1hr,3=1-2hrs, 4= Half a day, 5=A	*TMSPNT_CSC*

		whole day, *activated & mandatory for each answer in 55.18*	
OPTION, add another place, create type of place 2 etc,<br>Repeat 2.25-2.27 for each added place.			
Contacts, these contacts relate refer to only in the last 24-hours. From the time you/your child woke up yesterday to woke up today.			
55.21. Short contacts. How many people did you/your child see or have a short conversation with yesterday? (<5 min, exchange at least 5 words)	CSCSHTCONT	1= 0 – 9 people,2= 10 – 19 people,3= 20 – 29 people,4= >30 people, -99= Unknown; *mandatory*	*SHTCONT_CSC*
Close contacts, ≥ 5 mins, in the last 24-hours, from when you/your child woke up yesterday, to waking up today. (Contact with the same person at different times/locations needs to be recorded separately)			
55.22. Contact 1 Initials	CSCLNGCONTIN	XX, two letters, *change to capital, mandatory.*	*CLNGCONTIN _CSC*
55.23. Contact 1 Sex	CSCSXLNGCONT	0=Male, 1=Female, *activated & mandatory for each contact*	*SXLNGCONT _PCS*
55.24. Contact 1 Age	CSCAGLNGCONT	0-99*; activated & mandatory for each contact*	*AGLNGCONT _PCS*
55.25. Contact 1 Age Strata	CSCAGESTRLNGCT	0=Months 1=Years*; activated & mandatory for each contact*	*AGESTRLNGCT _PCS*
55.26. Type of contact with Contact 1	CSCCONTYPE	0=Non-physical, 1=Physical; *activated & mandatory for each contact*	*CONTYPE _PCS*
55.27. Relationship with Contact 1	CSCRELCONT	1=Household member, 2, Other relative, Colleague or Schoolmate, 3= Friend, 4= Client/Shop Owner, 5 = Other*; activated & mandatory for each contact*	*RELCONT _PCS*
55.28. Relationship with Contact 1, if other please specific.	CSCOTHRELCONT	Free text, *mandatory if 55.36=Other*	*OTHRELCONT _CSC*
55.29. Location of contact with Contact 1	CSCCTLOCA	1=Home, 2= Another House, 3=Work, 4=School, 5=Place of worship, 6=Public transport, 7=Market, 8= Field/Garden, 9=Other*; activated & mandatory for each contact*	*CTLOCA _CSC*
55.30. How often do you normally have contact with this person in	CSCFQCONT	1=Daily or almost daily,<br>2= At least once a week,	*FQCONT _CSC*

general?		3= At least once a month<br>4=Less than once a month<br>5= Never before*; activated & mandatory for each contact*	
55.31. What was the total amount of time you/your child spent with this contact?	CSCTOLCNTMSPNT	1=<15m, 2=15m- <1h, 3=1-<2h, 4= 2h-<4h, 5=≥4 hrs, -99=Unknown*; activated & mandatory for each contact*	*TOLCNTMSPNT_CSC*
[OPTION] ADD ANOTHER CONTACT, for each sequential contact, have option to add one more contact information, and remove if accidentally added another.<br>Repeat variables 55.22-> 55.31			
55.32. Estimate the total number of physical contacts your child had during a full school day today.	CSCPHYCONT	00-99, *activated & mandatory if 55.29=4*	*SCHCONT_CSC*
55.33. Staff Code	CSCPERSID	3 digits*; restricted to PCS staff &mandatory*	*PERSID_CSC*
